# COVID Challenges and Adaptations Among Home-Based Medical Practices: Lessons for an Ongoing Pandemic

**DOI:** 10.1093/geroni/igab046.2057

**Published:** 2021-12-17

**Authors:** Christine Ritchie, Orla Sheehan, Naomi Gallopyn, Shanaz Sharieff, Abraham Brody, Bruce Leff

**Affiliations:** 1 Massachusetts General Hospital, Boston, Massachusetts, United States; 2 Johns Hopkins University School of Medicine, Johns Hopkins University, Maryland, United States; 3 Massachusetts General Hospital, Boton, Massachusetts, United States; 4 NYU Hartford Institute for Geriatric Nursing, New York, New York, United States; 5 The Center For Transformative Geriatric Research, Johns Hopkins School of Medicine, Baltimore, Maryland, United States

## Abstract

Home-based primary care (HBPC) practices rapidly adapted to maintain care during the COVID-19 pandemic. This mixed-methods national online survey of HBPC practices probed responses to COVID-19 surges, COVID-19 testing, the use of telemedicine, practice challenges due to COVID-19, and adaptations to address these challenges. Seventy-nine practices across 29 states were included in the analyses. Eighty-five percent of practices continued to provide in-person care and nearly half cared for COVID-19 patients. Most practices also pivoted to concurrent use of video visits. The top five practice challenges were: patient familiarity with telemedicine, patient and clinician anxiety, technical difficulties reaching patients, and supply shortages. Practices also described creative strategies to physically support the needs of patients. These findings illustrate the need to balance in-person and virtual care for this population, and attend to the emotional needs of patients and staff.

